# Early Use of Intrapleural Tissue Plasminogen Activator and Dornase Alfa in Loculated Pleural Effusion Due to Mycobacterium Tuberculosis

**DOI:** 10.7759/cureus.49125

**Published:** 2023-11-20

**Authors:** Oluwafemi A Ajibola, Kyle I Happel

**Affiliations:** 1 Pulmonary and Critical Care Medicine, Louisiana State University Health Sciences Center, New Orleans, USA; 2 Medicine, Louisiana State University Health Sciences Center, New Orleans, USA

**Keywords:** tb, loculated tb pleural effusion, tuberculous pleuritis, intrapleural thrombolytic therapy, loculated pleural effusion, extrapulmonary manifestations of tuberculosis, mycobacterium tuberculosis, pleural effusion, mycobacterium tuberculosis (mtb)

## Abstract

Tuberculosis is a highly infectious respiratory disease due to *Mycobacterium tuberculosis* (MTb). The most common manifestation of MTb is pulmonary tuberculosis, but some patients can present with extrapulmonary manifestations as their initial presentation. Tuberculous pleurisy and pleural effusion are among the most common extrapulmonary manifestations of MTb. The treatment of pleural MTb is the same as the treatment for pulmonary disease, with a four-drug regimen with rifampin, isoniazid, pyrazinamide, and ethambutol (RIPE) under directly observed therapy (DOT). Drainage of the pleural effusion is usually not recommended in tuberculosis pleural effusion. We present a case of a complex, loculated pleural effusion due to MTb in an otherwise healthy middle-aged male who responded rapidly and completely to an early, short course of intrapleural tissue plasminogen activator and dornase alfa (TPA/DNase) therapy.

## Introduction

Tuberculosis is a highly infectious respiratory disease caused by the aerobic, gram-positive, acid-fast bacteria, *Mycobacterium tuberculosis* (MTb), which was first described by Robert Koch in 1882 [1]. Even though tuberculosis is both preventable and curable, it remains one of the leading causes of infectious death worldwide, responsible for 1.6 million deaths in 2021 [2]. Infection is transmitted by inhalation of infectious aerosol from individuals with pulmonary tuberculosis [1]. The most common manifestation of MTb is pulmonary tuberculosis, but up to 25% of adults present with extrapulmonary manifestations as their initial presentation [3]. Tuberculous pleurisy and pleural effusion are among the most common extrapulmonary manifestations of MTb, occurring in 3%-5% of patients. Some of the pleural effusions can form pockets of fluid due to fibrotic tissue from the inflammation, and when this occurs, it is called lobulated pleural effusion [4,5]. Tuberculous pleural effusion accounts for up to 80% of exudative effusions in developing countries but less than 1% in Western countries [3,5]. We present a case of a complex, loculated pleural effusion due to MTb in an otherwise healthy middle-aged male who responded rapidly and completely to an early, short course of intrapleural tissue plasminogen activator and dornase alfa (TPA/DNase) therapy by facilitating the breakdown of the loculation.

## Case presentation

**Figure 1:**
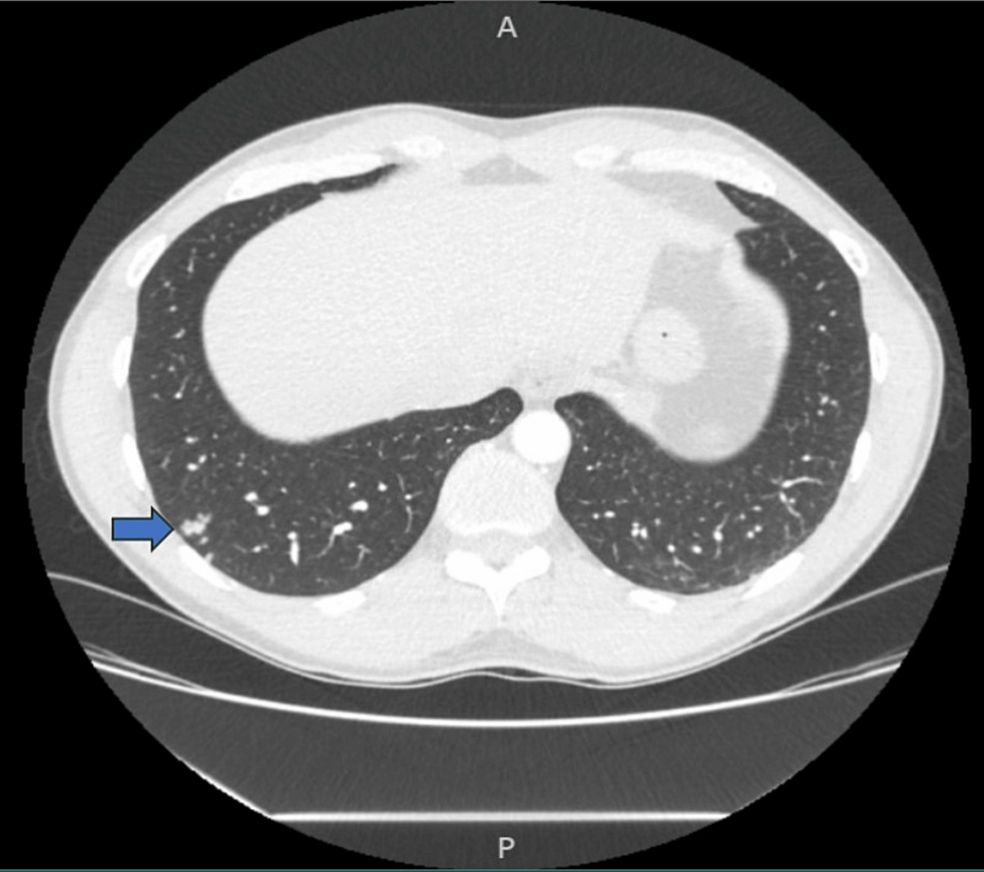
CT chest and abdomen with contrast show few small nodules in the right lower lobe. The blue arrow indicates the area of the small nodules.

**Figure 2:**
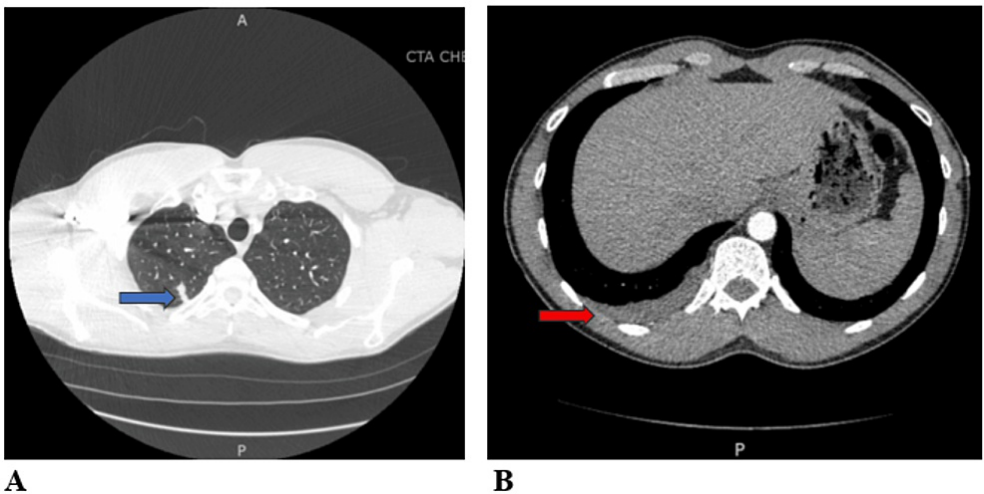
CT chest without contrast shows a cluster of tree-in-bud nodules in the right lower lobe, a new right upper lobe peripheral bandlike nodule, and a small right pleural effusion. The blue arrow indicates the right upper lobe peripheral bandlike nodule. The red arrow indicates the small right pleural effusion. (A) The lung window shows the right upper lobe peripheral bandlike nodule (blue arrow). (B) The soft tissue window shows a small right pleural effusion (red arrow).

A 42-year-old male with no significant past medical history presented with a one-day history of right-sided flank pain. The physical examination and routine lab work were unremarkable. CT chest and abdomen demonstrated a few small nodules in the right lower lobe (Figure 1). The patient was treated with azithromycin and ketorolac, with improvement in right-sided flank pain. The patient presented six months later with a two-day history of pleuritic right-sided lower chest pain, low-grade fever, headache, and diaphoresis. Repeat CT chest showed a cluster of tree-in-bud nodules in the right lower lobe, a new right upper lobe peripheral bandlike nodule, and a small right pleural effusion (Figure 2). The patient was again treated with azithromycin and Ibuprofen. The patient continued to have a right-sided pleuritic chest associated with a new dry cough, shortness of breath, and weight loss.

**Figure 3:**
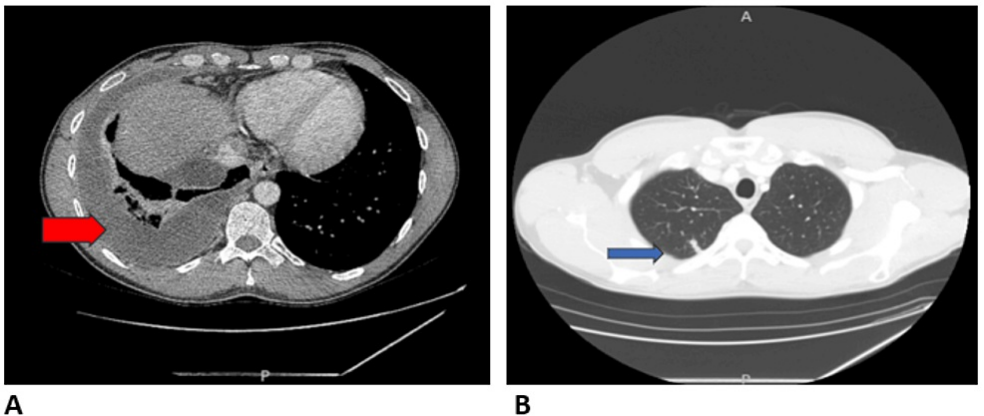
CT chest without contrast shows right pleural effusion and right upper lobe pulmonary nodule. The red arrow indicates right pleural effusion. The blue arrow indicates the right upper lobe pulmonary nodule. (A) The soft window shows the right pleural effusion (red arrow). (B) The lung window shows the right upper lobe pulmonary nodule (blue arrow).

A repeat CT scan one month later showed an enlarged right pleural effusion and right upper lobe pulmonary nodule (Figure 3). The patient underwent thoracentesis with immediate symptomatic improvement but developed recurrent symptoms one week later. The repeat CT demonstrated loculation of the right-sided pleural effusion. The patient was admitted to the hospital for IV antibiotics with ceftriaxone and azithromycin and underwent small-bore chest tube placement. The initial pleural fluid demonstrated a highly lymphocytic, exudative effusion (Table 1). Cytology was negative, and the acid-fast bacilli (AFB) stain showed no acid-fast bacilli. Intrapleural lytic therapy with TPA 10 mg and DNase 5 mg was given for three days due to incomplete drainage of the multiple loculations. With three days of twice-daily lytic therapy, complete evacuation of the pleural space was achieved, the pleural catheter was removed, and the patient was discharged home. While he was admitted, additional patient history was elicited, which revealed that he was deployed to West Africa for a few months three years prior as part of his military service. An interferon-gamma release assay (IGRA) returned positive, and a few weeks later, pleural fluid grew MTb complex. The patient was started on RIPE without recurrence of pleural effusion.

**Table 1:** Laboratory values WBC: White blood cells; LDH: Lactate dehydrogenase; AFB: Acid-fast bacilli; TB: Tuberculosis; /cu mm: /Cubic millimeter.

Labs	Latest reference range and units	Results
Pleural fluid color		Yellow
Pleural fluid appearance		Hazy
Pleural fluid WBC	/cu mm	5123
Segs, fluid	%	0
Lymphs, fluid	%	94
Monocytes/macrophages, fluid	%	6
Glucose, fluid	mg/dL	70
LDH, fluid	U/L	723
Body fluid, albumin	g/dL	3.2
Body fluid, protein	g/dL	5.4
Adenosine deaminase, pleural fluid	0–30 U/L	32
AFB culture stain pleural fluid		No acid-fast bacilli seen
TB gold plus	Negative	Positive
Culture		*Mycobacterium tuberculosis* complex
Cytology		Reactive lymphocytes (T-cells by immunohistochemical stains)

## Discussion

MTb pleuritis usually presents as an acute illness with fever, cough, and pleuritic chest pain. Patients may exhibit significant dyspnea when pleural effusion develops, particularly with large effusions [4]. Acute tuberculous pleuritis is usually seen in younger, immunocompetent individuals but can also occur in older individuals, especially in industrialized countries [4]. Other symptoms include night sweats, fatigue, weight loss, and chills [3,4,6,7]. Validated risk factors for extrapulmonary Mtb infection include young age, female gender, Asian and African origin, and human immunodeficiency virus (HIV) [6]. Without treatment, tuberculous pleural effusion can be complicated by pleural thickening, trapped lung, and fibrothorax, conditions that are associated with significant morbidity [7-9].

The pleural effusion of MTb is typically unilateral. Patients with MTb pleural effusion demonstrate parenchymal lung disease on chest radiographs in up to 20% of cases, while more than 80% exhibit lung findings on chest CT [7]. In patients with radiographic absence of pulmonary parenchymal MTb infection, tuberculosis pleural effusion is thought to occur either 6-12 weeks after primary infection or from more latent reactivation of tuberculosis. The latter is thought to be implicated more commonly in industrialized countries as is postulated in this case [7].

The pathogenesis of tuberculosis pleural effusion includes the rupture of subpleural caseous necrosis of the lung periphery into the pleural space [3,4,7]. This leads to the release of mycobacterial antigens into the pleural space and the activation of an intense inflammatory response in the pleural. The inflammatory response leads to increased production of pleural fluid and decreased fluid clearance due to impairment of lymphatic system drainage by the lymphocyte-rich pleural fluid. Although initial inflammatory cells in the effusion are neutrophils in the first two weeks, these are replaced by lymphocytes. The lymphocytic exudate is later accompanied by pleural granuloma formation and the release of adenosine deaminase into pleural fluid (ADA) [3].

Maintaining clinical suspicion for MTb is key to establishing the diagnosis of pleural tuberculosis. Our patient was treated with antibiotics multiple times for community-acquired pneumonia. In retrospect, the initial CT demonstrated a small, lower lobe subpleural nodule, an unusual finding for community-acquired pneumonia (CAP). The subsequent development of pleural effusion and peripheral upper lobe nodule despite antibiotic therapy was also unusual for CAP in an otherwise healthy young adult.

The diagnosis of pleural tuberculosis is based on the detection of MTb in pleural fluid, sputum, or pleural biopsy specimens by culture or the presence of caseating granulomas along with acid-fast bacilli on histologic specimen from pleural biopsy [3]. Unfortunately, the sensitivity of pleural fluid AFB stain is low, identifying organisms in fewer than 10% of cases. This increases to roughly 20% in patients with HIV and tuberculosis empyema and to 52% when staining induced sputum [3,6,10]. The use of liquid culture media instead of solid culture media has improved culture sensitivity and decreased culture time [3,6,10].

In MTb-endemic regions, the diagnosis of pleural tuberculosis is readily established based on clinical suspicion combined with a lymphocytic, exudative pleural effusion as aided by biomarkers such as pleural fluid adenosine deaminase [7]. In the early stage (less than two weeks) of the disease, polymorphonuclear cells may be the predominant inflammatory cells [3,6,7,10].

In low MTb prevalence regions, the diagnosis of pleural tuberculosis is often delayed due to low clinical suspicion, absence of typical imaging findings, suboptimal social/occupational history taking, low sensitivity of “usual” pleural fluid diagnostic studies, and the slow growth of the organism [6]. The utility of pleural fluid adenosine deaminase varies with the prevalence of tuberculosis as low pleural fluid adenosine deaminase levels help to exclude pleural tuberculosis in non-endemic regions. Other tests that can be done on the pleural fluid include IFN-gamma and MTb nucleic acid amplification test (NAAT), although the former is not widely available for pleural fluid and does not provide a definitive diagnosis of extrapulmonary tuberculosis disease, while the latter is associated with low sensitivity [10]. Fortunately, patients with isolated tuberculosis pleural effusion do not need to be isolated unless sputum is smeared or culture-positive for MTb [7].

The treatment of pleural MTb is the same as the treatment for pulmonary disease, with a four-drug regimen of rifampin, isoniazid, pyrazinamide, and ethambutol (RIPE) under directly observed therapy (DOT) [11]. Drainage of the MTb pleural effusion is not recommended unless needed for symptom relief. The effusion typically resolves within 6-12 weeks, and routine drainage does not appear to reduce the risk of residual pleural thickening that could occur in up to 50% of patients. For instance, a prior study found no benefit to the routine addition of pigtail drainage to anti-MTb drugs in tuberculous effusions [12]. We believe the situation is different for MTb effusions that present loculated. A prior randomized control trial supported the use of intrapleural streptokinase in the treatment of loculated tuberculous pleural effusion [13]. Our patient was treated with the more contemporary three-day course of tPA/DNase as was used in the well-referenced MIST2 study for complicated parapneumonic effusions [14]. The fact that our patient experienced near complete resolution of his loculated tuberculous pleural effusion supports the concept that fibrinolytic therapy is warranted in such cases of early detected loculated tuberculous effusions. This is an important point as early complete drainage has been shown to correlate better with symptom recovery than any subsequent therapy [15]. Regardless of fibrinolytic use, patients initiated on treatment should be monitored for paradoxical worsening of pleural effusion, which has been described in the literature [6,7].

## Conclusions

Clinical suspicion and thorough history taking are essential in diagnosing pleural tuberculosis in low-endemic areas like the United States. Early fibrinolytic therapy for loculated MTb effusion speeds the resolution of pleural disease, facilitates rapid symptom improvement, and decreases the need for subsequent pleural intervention.

## References

[1] (2023). History of world TB day. https://www.cdc.gov/tb/worldtbday/history.htm.

[2] (2023). Tuberculosis. https://www.who.int/en/news-room/fact-sheets/detail/tuberculosis.

[3] Vorster MJ, Allwood BW, Diacon AH, Koegelenberg CF (2015). Tuberculous pleural effusions: advances and controversies. J Thorac Dis.

[4] Jeon D (2014). Tuberculous pleurisy: an update. Tuberc Respir Dis (Seoul).

[5] Udwadia ZF, Sen T (2010). Pleural tuberculosis: an update. Curr Opin Pulm Med.

[6] Lee JY (2015). Diagnosis and treatment of extrapulmonary tuberculosis. Tuberc Respir Dis (Seoul).

[7] Light RW (2010). Update on tuberculous pleural effusion. Respirology.

[8] Shaw JA, Koegelenberg CF (2021). Pleural tuberculosis. Clin Chest Med.

[9] Shaw JA, Irusen EM, Diacon AH, Koegelenberg CF (2018). Pleural tuberculosis: a concise clinical review. Clin Respir J.

[10] Lewinsohn DM, Leonard MK, LoBue PA (2017). Official American Thoracic Society/Infectious Diseases Society of America/Centers for Disease Control and Prevention Clinical Practice Guidelines: diagnosis of tuberculosis in adults and children. Clin Infect Dis.

[11] Nahid P, Dorman SE, Alipanah N (2016). Official American Thoracic Society/Centers for Disease Control and Prevention/Infectious Diseases Society of America Clinical Practice Guidelines: treatment of drug-susceptible tuberculosis. Clin Infect Dis.

[12] Lai YF, Chao TY, Wang YH, Lin AS (2003). Pigtail drainage in the treatment of tuberculous pleural effusions: a randomised study. Thorax.

[13] Chung CL, Chen CH, Yeh CY, Sheu JR, Chang SC (2008). Early effective drainage in the treatment of loculated tuberculous pleurisy. Eur Respir J.

[14] Rahman NM, Maskell NA, West A (2011). Intrapleural use of tissue plasminogen activator and DNase in pleural infection. N Engl J Med.

[15] Wyser C, Walzl G, Smedema JP, Swart F, van Schalkwyk EM, van de Wal BW (1996). Corticosteroids in the treatment of tuberculous pleurisy. A double-blind, placebo-controlled, randomized study. Chest.

